# Does the pain sensitivity questionnaire correlate with tourniquet pain in patients undergoing ankle surgery?

**DOI:** 10.3389/fsurg.2023.1102319

**Published:** 2023-02-27

**Authors:** Qiuyue Fu, Mingming Han, Yuyang Mu, Lina Hao, Liang Lu, Xiang Huang, Juan Li, Fang Kang

**Affiliations:** ^1^ Department of Anesthesiology, The First Affiliated Hospital of University of Science and Technology of China, Hefei, Anhui, China; ^2^ Department of Hand and Foot Surgery, The First Affiliated Hospital of University of Science and Technology of China, Hefei, Anhui, China

**Keywords:** pain sensitivity questionnaire, tourniquet, ankle surgery, nerve block, pain

## Abstract

**Background:**

Tourniquet pain is the most prominent problem in ankle surgery, and there is no proper method to predict it. It was reported that pain sensitivity questionnaires could evaluate the pain sensitivity of subjects. Its potential to predict tourniquet pain in ankle surgery is constructive and meaningful.

**Methods:**

One hundred and twenty patients undergoing ankle surgery were included in this study. The pain sensitivity questionnaire (PSQ) and self-rating anxiety scale (SAS) were completed before the operation. The methods included an ultrasound-guided popliteal sciatic, a femoral nerve block, and a proximal thigh tourniquet. The pressure of the tourniquet was set according to the systolic blood pressure (SBP + 100 mmHg). A visual analogue scale (VAS) was used to assess the tourniquet pain. Also, the onset time of tourniquet pain ≥4 VAS units was recorded.

**Results:**

The PSQ-total and PSQ-minor scores were significantly correlated with the onset time when the tourniquet pain ≥4 VAS units (*r* = −0.763, *r* = −0.731, *P* < 0.001). The PSQ-total score <6.5 group gave significantly lower ratings for items 3, 4, 14, and 16 in the PSQ survey compared to the PSQ-total score ≥6.5 group (*P* < 0.05). Patients with high pain sensitivity have a higher need for analgesic drugs (*P* < 0.001). PSQ-total score ≥6.5 (OR = 185.8, 95% CI = 39.8–1,437.6, *P* < 0.001), sex (male, OR = 0.11, 95% CI = 0.018–0.488, *P* < 0.05), and age (OR = 0.92, 95% CI = 0.842–0.995, *P* < 0.05) were risk factors for reporting a tourniquet pain ≥4 VAS units within 30 min.

**Conclusion:**

The PSQ score is found to be correlated with intraoperative tourniquet pain. In addition, sex and age also affect the time of having intraoperative tourniquet pain.

## Introduction

A proximal thigh tourniquet is often used in foot and ankle surgery to facilitate a bloodless field. With the extended use of a tourniquet, patients often complain of pain and discomfort at the tourniquet site or distal limb (1). However, it is difficult to relieve tourniquet pain, and the sensitivity of different patients to tourniquet pain is different. So, predicting the sensitivity to tourniquet pain might help in choosing a reasonable anesthesia scheme. Numerous studies have tried to predict acute postoperative pain by experimental pain tests or quantitative sensory tests (QSTs) before surgery (2, 3). None of these methods have been used as a routine approach for predicting pain due to the absence of an instrument and/or being time-consuming. The pain catastrophizing scale (PCS) describes different feelings and thoughts associated with pain and is used routinely in some preoperative settings for all patients (4). However, the PCS is a cognitive and emotional tendency and is not based on life events. The pain sensitivity questionnaire (PSQ) is a pain sensitivity self-assessment questionnaire based on events in daily life and designed by Ruscheweyh et al. (5). Furthermore, the PSQ is validated in Chinese populations and can typically be completed in less than 15 min, without the need for any special equipment or staff. Moreover, the questionnaire does not have any ethical concerns. Prior studies have shown that the PSQ can evaluate the pain sensitivity of subjects, predict postoperative acute pain, and screen individuals at high risk of postoperative chronic pain (6). However, whether the PSQ can predict the sensitivity of patients to tourniquet pain has not been reported as yet.

This study aims to evaluate the feasibility of the PSQ in predicting tourniquet pain in ankle surgery. Preoperative PSQ evaluation is helpful for guiding the formulation of an anesthesia plan, intervening in advance for predictable pain, and implementing individualized anesthesia management.

## Materials and methods

### Participants

The trial was registered at www.Chictr.org.cn on 9 July 2021 prior to patient recruitment (code: ChiCTR2100048525). The clinical trial was approved by the Medical Research Ethics Committee of the First Affiliated Hospital of the University of Science and Technology of China (ethics: 2020KY). The study was conducted between July 2021 and February 2022. Written informed consent was obtained from all participants. Patients aged ≥18 years with ASA (American Society of Anesthesiologists physical status classification) I and II and scheduled for ankle and calcaneal fracture surgery were recruited. Exclusion criteria were as follows: inability to consent to the study, multiple sclerosis, sensory loss, or other diseases affecting sensory function such as diabetes, receiving major opioids for chronic pain therapy or substance abuse, narcotic-intolerant, hepatic or renal failure, allergy to local anesthetics, pregnancy, and prior surgery in the popliteal fossa.

### Preoperative assessment

The day before surgery, all patients were asked to complete the Chinese version of the PSQ (5, 7) and SAS (8) independently or with the assistance of researchers. The validity of the Chinese version of the two inventories has been confirmed (7). According to previous studies, the enrolled patients were assigned to either a low (PSQ score <6.5) or a high PSQ group (PSQ score ≥6.5) (9). The PSQ has 17 questions about life situations associated with pain, of which three are normally considered not painful by healthy subjects and are therefore excluded from the final score. The PSQ-total score was the average rating of items 1, 2, 3, 4, 6, 7, 8, 10, 11, 12, 14, 15, 16, and 17 (all but the three non-painful items), and the PSQ-minor score was the average rating of items 3, 6, 7, 10, 11, 12, and 14. The PSQ assesses individual pain sensitivity by imagining the pain degree of some scenes in life and performing VAS pain scores. The SAS is divided into 20 items (1–4/item), and a total score of 40–49 indicates mild anxiety, 50–59 moderate anxiety, ≥59 severe anxiety, and ≥40 anxiety patients.

### Anesthesia and data collection

After entering the operating room, participants had intravenous cannulas (20-G) inserted into the peripheral vein of the upper limb, and 500 ml sodium acetate Ringer solution was intravenously injected within 30–40 min. The standardized monitoring methods of electrocardiogram (ECG), oxygen saturation (SPO_2_), and noninvasive blood pressure were established. All patients received intravenous dexmedetomidine sulphate 0.5 μg/kg within 15 min. All patients were placed in a lateral decubitus position, with the operated limb on the upper side and the hip and knee slightly bent. The block of the sciatic nerve in the popliteal fossa was performed under sterile conditions by ultrasound guidance (sonosite edge, USA). A total of 25 ml of a mixed solution of 0.25% ropivacaine and 1% lidocaine was injected around the sciatic nerve and adequate spread of the drugs around the nerves was monitored. Then, in the supine position, 10 ml of the same solution was injected for the femoral nerve block. In addition, adequate spread of the drug around the nerves was monitored. All blocks were performed by the same anesthesiologist. The level of sensory block was assessed using the ice test in 0–2 scale every 5 min. Sensory block rating: 0 = complete loss of sensation; 1 = hypoesthesia; 2 = feeling normal. Successful surgical block was defined as a grade 0 sensory block within 30 min (10). If the sensory block level was greater than 0 at 30 min, or the patient complained of pain at the time of skin incision, the patient was excluded from the study. When a complete sensory block was achieved, a 9 cm × 60 cm wide pneumatic thigh tourniquet (Changzhou Yanling Electronic Equipment Co., Ltd., model: ats-I) was applied to the proximal part of the thigh. The pressure was set at 100 mmHg more than SBP and the duration was 90 min. When the duration of tourniquet inflation was longer than 2 h, deflation of the tourniquet was performed for 10–15 min to allow tissue reperfusion (11). The tourniquet pain intensity was assessed by a blinded observer immediately after tourniquet inflation (time 0), every 5 min up to 20 min, and then every 15 min until the end of surgery with a 10-point visual analogue scale (VAS) (0 = no pain, and 10 = unbearable pain). Mostly, VAS ≤ 4 units was taken as mild pain and a widely acceptable pain threshold for patients (12, 13). If the patient complained of thigh pain and VAS ≥ 4 units, 5 µg of sufentanil was given intravenously. Additional 5 µg of sufentanil was given in the case of insufficient pain relief 5 min later. If thigh pain was still unbearable, general anesthesia was established. The main outcome is the onset time of tourniquet pain ≥4 VAS units. Persistent low limb numbness/paresthesia or motor deficit were also recorded within 48 h after the surgery.

### Sample size and statistical analysis

The sample size was based on a pilot experiment of 20 cases in our observation. Also, the correlation coefficient between PSQ-total and the onset time of reporting a tourniquet pain score ≥4 VAS units was approximately −0.35. Using a power of 90% and a two-sided alpha error of 0.05, 83 participants were needed. Considering a 10% dropout rate, 90 participants were needed.

SPSS 26 was used for statistical analysis. Normally distributed continuous variables were expressed as the mean ± SD and compared between groups using a two-sample Student's *t* test. If the distribution was not normal, the median with interquartile range (IQR) was expressed, and a *χ*^2^ test was used. Pearson correlation analysis was used for data conforming. Logistic regression analysis was used to predict the risk factors for reporting tourniquet pain ≥4 VAS units within 30 min. All *P-*values < 0.05 were considered to be statistically significant.

## Results

A total of 123 patients undergoing ankle and heel fracture surgery were enrolled in the study. Three patients who complained of pain at the moment of skin incision were excluded. Finally, the remaining 120 patients completed the protocol. The demographic characteristics of patients are listed in Table 1.

**Table 1:** Patients’ demographic characteristics and perioperative data (*N* = 120).

Age (years)	39 ± 17
Gender (M/F)	83/37
BMI (kg/m^2^)	24.6 ± 0.32
Educational background	
High school degree or below	52
Above high school degree	68
Tourniquet inflation duration (min)	55 ± 20

The PSQ-total score of all patients was 5.58 ± 1.44, ranging from 1.9 to 9.1. The number of patients with PSQ-total <6.5 was 80, and the number of other patients was 40. The PSQ-minor score of all patients was 2.42 ± 0.85, ranging from 0.8 to 4.6. The onset time of tourniquet pain ≥4 VAS units was 38 ± 17.3 min. Thirty-nine patients reported tourniquet pain ≥4 VAS units within 30 min, and others reported tourniquet pain 30 min later.

**Figure 1:**
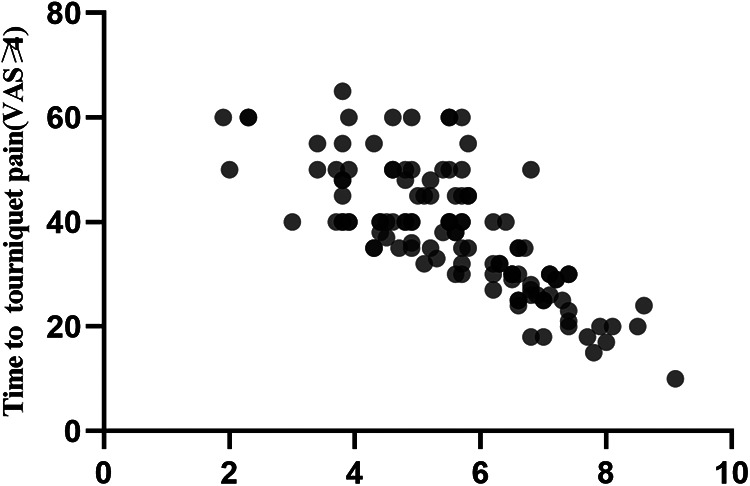
Scatter plot of PSQ-total and tourniquet pain score ≥4 VAS.

**Figure 2:**
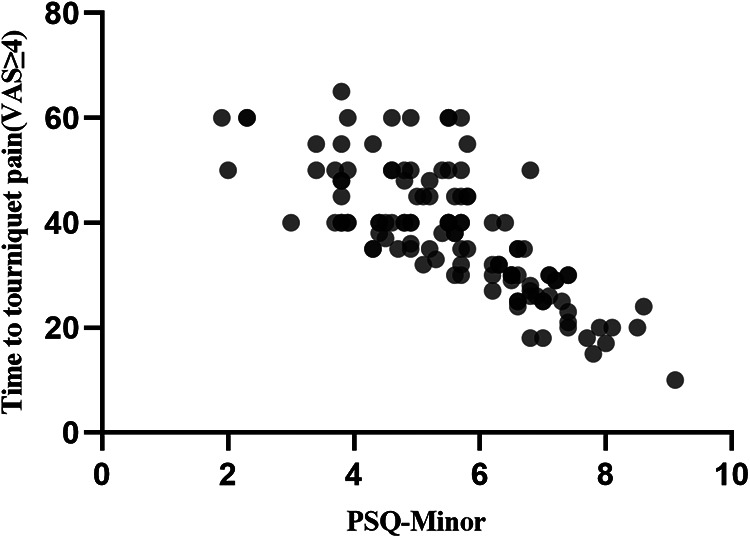
Scatter plot of PSQ-minor and tourniquet pain score ≥4 VAS.

The PSQ-total and PSQ-minor scores were found to be significantly correlated with the onset time of tourniquet pain score ≥4 VAS (*r* = −0.763, *r* = −0.731, *P* < 0.001; Figures 1,2).

The PSQ-total score <6.5 group gave significantly lower ratings for items 3, 4, 14, and 16 in the PSQ survey compared to the group with a PSQ-total score ≥6.5 (*P* < 0.05). The ratio of tourniquet pain VAS ≥ 4 units at 30 min and the total amount of additional remedial sufentanil were significantly greater in the PSQ-total score ≥6.5 group compared with the PSQ-total score <6.5 group (*P* < 0.05). The number of patients who required general anesthesia was not significantly different between the two groups (Table 2).

**Table 2:** Comparison of perioperative conditions between the two groups of patients.

	PSQ scores <6.5 (*n* = 80)	PSQ scores ≥6.5 (*n* = 40)	*P-*value
Score of item 3	2.86 ± 1.41	4.35 ± 1.23	<0.001
Score of item 4	4.41 ± 1.71	6.38 ± 1.61	<0.001
Score of item 14	1.69 ± 0.98	2.28 ± 2.05	<0.05
Score of item 16	3.25 ± 1.84	5.6 ± 2.39	<0.001
Tourniquet pain ≥4 VAS units within 30 min	4	35	<0.05
Amount of sufentanil used (μg)	60	110	<0.001
Number of conversions to general anesthesia	7	5	0.475

The results of the logistic regression analysis are presented in Table 3. PSQ-total score ≥6.5, sex, and age were risk factors for reporting a tourniquet pain ≥4 VAS units within 30 min (*P* < 0.05). The SAS score of all patients was 23 ± 3. Also, no patient was anxious before the surgery.

**Table 3:** Logistic regression analysis of reporting tourniquet pain ≥4 VAS units within 30 min.

	OR	95% CI	*P*-value
PSQ-total ≥6.5	185.8	39.8–1,437.6	<0.001
Male	0.11	0.018–0.488	0.006
Age	0.92	0.842–0.995	0.045
BMI	1.04	0.871–1.238	0.678
Above high school degree	0.38	0.007–1.800	0.228
SAS	0.85	0.707–1.008	0.051

None of the subjects enrolled in the study had complications or side effects associated with the nerve block or tourniquet-related pain within 48 h after surgery.

## Discussion

This study investigated the correlation between the PSQ and tourniquet pain to evaluate whether it could identify patients susceptible to tourniquet pain and provide a basis for improved clinical management through treatment strategies in each patient.

We enrolled patients with ankle or heel fractures; thus, the femoral nerve and the sciatic nerve block could meet the analgesia requirement of the procedure. The effectiveness of different approaches to sciatic nerve block in preventing tourniquet pain is controversial. Spasiano et al. reported that the combination of proximal sciatic nerve block with femoral nerve and lateral femoral cutaneous nerve block might greatly suppress thigh pain after tourniquet inflation (14). In contrast, Fuzzier et al. found that the proximal approach of the sciatic nerve block provides no better thigh tourniquet pain relief than the popliteal approach during foot surgery (15). Furthermore, the popliteal fossa approach was easy to carry out, resulting in less discomfort postoperatively than the posterior approach because the nerve is more superficial and does not affect the movement of the thigh. Thus, the popliteal fossa approach of the femoral nerve and sciatic nerve block was established in this study.

We found that both PSQ-total and PSQ-minor scores are negatively correlated with the onset time of tourniquet pain ≥4 VAS units, which means that the PSQ score and its subgroup could predict the tourniquet pain to some extent. In healthy subjects, experimental pain intensity rating scores were more strongly correlated with the PSQ-minor subscore than with the PSQ-total score. Nevertheless, the authors mostly recommend that both the PSQ-total score and the PSQ-minor subscore should be collected (5, 6). Bearing this in mind, we used both PSQ-total and PSQ-minor in our study and observed similar trends in their correlation with the onset time of tourniquet pain ≥4 VAS units. Previous studies also demonstrated the similar strength of these two scores in patients with chronic pain (4, 16). Another finding is that patients with PSQ-total score ≥6.5 were more likely to report tourniquet pain earlier in the procedure and needed more rescue analgesics. These results indicated that patients with high PSQ are more sensitive to tourniquet pain. Tourniquet pain is described as a dull, tight, aching sensation at the site of tourniquet application. So, we analyzed several items (3, 4, 14, 16) of PSQ that were similar to the pain type of tourniquet and found that items 3, 4, 14, 16 scores were significantly lower in the PSQ-total score <6.5 group than those in the PSQ-total score ≥6.5 group. Furthermore, Duchow et al. also found that patients with high PSQ scores were more prone to central sensitization and more sensitive to acute and chronic pain (17). Accordingly, spinal or general anesthesia should be considered in patients with high PSQ scores instead of peripheral nerve blocks alone. The validity of PSQ in predicting pain sensitivity and intensity of postoperative pain has been confirmed in several studies. Bjornnes et al. reported a positive correlation between the PSQ score and acute postoperative pain intensity in patients undergoing abdominal, pulmonary, thyroid, and cardiac surgeries (17, 18). Moreover, the PSQ score affects improvements in pain and disability after spine surgery (9). Patients with higher PSQ have less improvement in postoperative back pain, leg pain, and disability. However, our findings showed that seven patients in the PSQ-total score <6.5 group required general anesthesia during the surgery, suggesting that tolerance of tourniquet pain is not only correlated with the PSQ score alone but also with other factors.

Another finding of our study is that female gender and age, besides PSQ, were all risk factors for the occurrence of tourniquet pain ≥4 VAS units within 30 min. Our results indicated that women might report tourniquet pain earlier than men after the tourniquet is inflated. Population-based pain research has shown that women are more likely than men to report a variable range of temporary and persistent pains and to report more severe pain, more frequent pain, and pain of longer duration than men (19–21), which is consistent with our study. In addition, older patients may report tourniquet pain later compared to younger patients. Also, previous studies had found lower subjective pain scores when older patients received painful stimuli, presumably related to reduced sensory nerve function in the elderly (22).

In this study, we tried to detect whether anxiety was a risk factor for pain sensitivity. Unexpectedly, we found that the SAS scores of all patients were less than 40, which means no patient was anxious before the surgery. Perhaps this was because all the subjects thought the procedure would be safe and were full of confidence about the curative effect.

There are some limitations in the present study. We did not measure the thigh circumference and local fat thickness of the tourniquet, which may affect the patient's susceptibility to tourniquet pain.

## Conclusion

In conclusion, PSQ scores are found to be associated with intraoperative tourniquet pain. In addition, patients with high PSQ scores, women, and younger patients may have an earlier onset of tourniquet pain. Moreover, preoperative PSQ assessment can help guide the development of anesthetic protocols, earlier intervention for predictable pain, and the implementation of individualized anesthetic management.

## Data Availability

The data analyzed in this study is subject to the following licenses/restrictions: Involve patient privacy. Requests to access these datasets should be directed to *709765934@qq.com*.
